# CRISPR-Cas Systems and Genome Editing: Beginning the Era of CRISPR/Cas Therapies for Humans

**DOI:** 10.3390/ijms25105292

**Published:** 2024-05-13

**Authors:** Dmitry S. Karpov

**Affiliations:** Center for Precision Genome Editing and Genetic Technologies for Biomedicine, Engelhardt Institute of Molecular Biology, Russian Academy of Sciences, Vavilov Str. 32, Moscow 119991, Russia; aleom@eimb.ru

Harnessing of CRISPR/Cas (Clustered Regularly Interspaced Short Palindromic Repeats/CRISPR-associated genes) systems for detection, chemical modification, and sequence editing of nucleic acids dramatically changed many fields of fundamental science, biotechnology, and biomedicine [1,2]. No wonder that the inventors of technology, Emmanuelle Charpentier and Jennifer Doudna, have been awarded the Nobel Prize in 2020 [3]. Natural CRISPR/Cas systems are well suited to disrupting coding or non-coding sequences in the genome, are much less effective at distinguishing and editing sequences with single-nucleotide differences, and are prone to recognizing similar sequences in the genome [4]. Therefore, further exploration of the CRISPR/Cas world continues both to find new genome and epigenome editors and to engineer known editors to overcome their shortcomings to make their applications more efficient, predictable, and safe.

The aim of this Special Issue is to gather knowledge on the current state of the rapidly growing field of research and application of CRIPSR/Cas systems and other genome editing tools in basic science, biotechnology, and biomedicine. The applications of CRISPR/Cas systems discussed in the articles in this Special Issue are summarized in Figure 1.

Currently, complete CRISPR/Cas systems are found in more than 12,600 strains of bacteria and archaea, according to CRISPRCasDB [5]. This is less than 1% of known bacterial and archaea strains (402,709 strains with complete genomes), according to the Genome Taxonomy Database [6]. Thus, the prokaryote universe provides an opportunity for a broad search for novel genome editors. The greatest efforts are focused on exploring the biotechnological potential of small-sized Cas effectors. Since Cas9 from Streptococcus pyogens is the most active genome editor both in vitro and in vivo [7,8], smaller Cas9 orthologs are preferred. Thus, AnoCas9 from the thermophilic bacterium Anoxybacillus flavithermus, characterized in vitro by Matveeva et al., provides a good example of such small Cas effectors. AnoCas9 is a 1087 aa protein that exhibits nuclease activity in the 37–60 °C range and a PAM preference for 5′-NNNNNCDAA-3′ in vitro. This Cas protein is a valuable addition to the known set of thermophilic Cas proteins used, for example, for enrichment of sequencing libraries, allele-specific isothermal PCR, detection of human viruses, etc. [9,10].

CRISPR/Cas systems have revolutionized the field of biotechnology. The traditional yeast Saccharomyces cerevisiae has long been used to create improved producers of valuable substances because of its highly active homologous recombination, which allows the reconstruction of entire heterologous biochemical pathways [11]. In the shadow of *S. cerevisiae*, it was much less known and very difficult to genetically engineer nonconventional yeasts with high biotechnological potential. Genetic engineering of nonconventional yeasts has mainly relied on random mutagenesis using UV light, chemical mutagens, or transposons [12], followed by the laborious selection of mutant strains with desired properties. Xia et al., in their comprehensive review, describe the application of various CRISPR/Cas-based approaches for metabolic engineering of both conventional and nonconventional yeasts [13]. The authors also point out the drawbacks of CRISPR/Cas-based systems that should be considered when planning genome editing experiments in yeast. The development of CRISPR/Cas approaches for plant genome engineering [14] takes plant biotechnology to a new level. Fast-growing poplar trees have good potential in the paper industry, biofuel production, biomedicine, urban greening, and soil bioremediation. In their comprehensive review, Kovalev et al. describe the currently known molecular mechanisms of poplar immune defense as well as various ways, including genome editing using CRISPR/Cas systems, to improve poplar resistance to pathogens.

In addition to biotechnological applications, CRISPR/Cas systems are widely used in biomedicine, for example, to create cellular and animal models of human diseases [15]. Knockout animals are commonly used to study the mechanisms of pathogenesis associated with the gene of interest [16]. McBeath et al. detail an approach using CRISPR/Cas9, called recombination-regulated artificial intron (rAI), to generate conditional knockout mice. The rAI method is simpler, faster, and cheaper than currently known methods for tissue-specific knockout in mice. In another interesting intron-related story, Matveeva et al. used CRISPR/Cas9 editing of introns within the tumor-suppressive long non-coding RNA GAS5 to discover a quite interesting epigenetic mechanism for processing this lncRNA [17]. The authors also obtained mutant cell lines with the deletion of a significant portion of GAS5. Transcriptome analysis of mutant cell lines revealed the involvement of lncRNA GAS5 in the regulation of genes related to cell adhesion, signal transduction, and cell-membrane structures. CRISPR/Cas systems are often used to create cellular models of human diseases, including complex neuropsychiatric diseases such as schizophrenia (SZ). Abashkin et al. found that the gene encoding the transcription factor ASCL1 is associated with SZ [18]. Using CRISPR/Cas9, the authors created a mutant SH-SY5Y cell line with a functional knockout of ASCL1, and transcriptome analysis of the mutant cell line allowed them to identify genes whose deregulation by ASCL1 dysfunction may be associated with the depletion of GABAergic neurons and/or reduced neuroplasticity. Indeed, these pathogenic processes are observed in the brains of patients with SZ [19,20].

In animal models as well as in therapeutic approaches, CRISPR/Cas systems are used more often for gene knockout than for knock-in because of weak homologous recombination in mammalian cells [21]. Leal et al. comprehensively discuss current approaches to enhance homologous recombination in CRISPR/Cas9-based gene therapy applications [22].

There are several ways to deliver the CRISPR/Cas system into mammalian cells, and the ribonucleoprotein (RNP) complex provides a fast, efficient, less cytotoxic, and more specific way to edit the genome [23,24]. Therefore, purification technologies are needed to reconstitute RNP complexes. Due to its large size and high cytotoxicity to bacterial cells when overexpressed [25], SpCas9 is a difficult protein to purify from bacterial hosts. Evmenov et al. described efficient protocols for the expression and purification of SpCas9 from *E. coli*. In addition, they compared several delivery methods for the CRISPR/Cas system and found that the RNP complex combined with a transfection reagent allows for more efficient editing in mammalian cells compared to plasmid transfection or incubation with RNP. Increasing the stability and specificity of sgRNAs can significantly improve the efficiency of genome editing in mammalian cells [26,27]. It is known that sgRNA stabilization can improve the efficiency of genome editing. In addition to altering sgRNA structure, a number of chemical modifications of sgRNAs have been proposed [28,29]. Prokhorova et al. propose to chemically modify sgRNA with N1-methylpseudouridine as a way to stabilize sgRNA. This modification does not affect Cas9 activity in targets but decreases off-target activity.

Naturally, prokaryotes are using CRISPR/Cas systems to target their viruses [30]. Therefore, it is rational to apply CRISPR/Cas systems to treat human viral diseases. The prevalence of drug-resistant strains of human viruses [31,32,33] limits treatment options. Globally distributed herpesviruses and hepatitis viruses are of greatest concern because of the burden of disease and death they cause and the potential for outbreaks and epidemics [34,35]. These viruses have a latent form in their infectious cycle [36,37] that is resistant to known therapeutic agents. Moreover, latent human or animal viruses can contribute significantly to graft rejection in transplantation [38] or xenotransplantation [39]. Therefore, alternative, effective, and safe strategies to combat human viruses are urgently needed. In this Special Issue, Bartosh et al. discuss novel CRISPR/Cas-based strategies for the treatment of hepatitis viruses. In another study, Karpov et al. obtained promising results on effective suppression of herpes simplex virus type 1 replication using CRISRPR/Cas9 and CRISPR/CasX systems in Vero cells. Another way to combat human viruses is through the development of vaccines. In the case of influenza viruses, traditional technology based on culturing the virus in chicken embryos has several drawbacks, including long incubation times (6 months), allergic reactions to egg components, occasional antigenic mismatch between egg-adapted viruses, and challenges in obtaining avian pandemic strains [40]. Therefore, alternative methods of rapid and safe production of influenza vaccines are needed. Eshchenko et al. described the creation of the human cell line WI-38 VA13 with a knockout of the gene encoding interferon-inducible transmembrane protein 3, which prevents the virus from entering the cell.

Genome editing technology based on CRISPR/Cas systems gives humanity hope for curing not only viral but also hereditary diseases. However, CRISPR/Cas-based molecular tools have a number of drawbacks that limit their therapeutic applications. Hryhorowicz et al. comprehensively reviewed these drawbacks and discussed the development of improved genome editors for more efficient and safer applications in human gene therapy [41]. Karpov et al., in their comprehensive review, consider current approaches to the treatment of type 1 diabetes using cellular regenerative medicine approaches, with a focus on CRISPR/Cas-engineered cell products [42]. Special attention is given to the possible drawbacks of cellular and CRISPR/Cas-based technologies and how to overcome them. Paschoudi et al. highlight the recent successes of therapeutic approaches utilizing CRISPR/Cas-based genome editing to treat β-hemoglobinopathies, including sickle cell disease [43]. Notably, in late 2023, the FDA approved a cell-based gene therapy called CasgevyTM for the treatment of sickle cell disease. CasgevyTM is the first FDA-approved therapy utilizing CRISPR/Cas9-based genome editing technology [44]. Thus, ten years after the first application of the CRISPR/Cas9 system for genome editing in mammalian cells, presented in three seminal papers [45,46,47], the first CRISPR/Cas9-based cellular product for curing human disease opens the era of CRISPR/Cas therapies for humans.

## Figures and Tables

**Figure 1:**
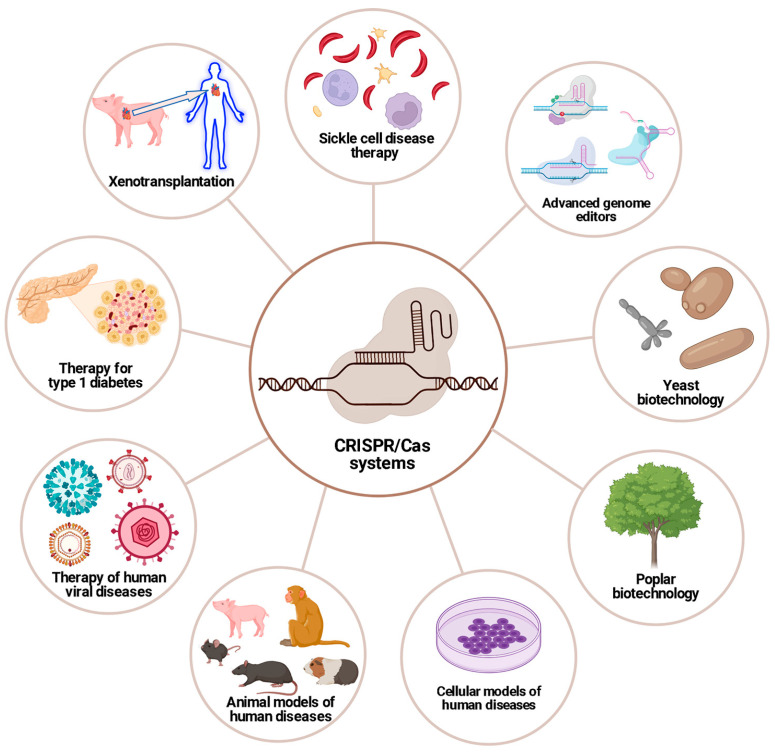
Selected applications of CRISPR/Cas-based genome editing that are discussed in the Special Issue. Created with BioRender.com.

## Data Availability

Not applicable.

## References

[1] Ding W., Zhang Y., Shi S. (2020). Development and Application of CRISPR/Cas in Microbial Biotechnology. Front. Bioeng. Biotechnol..

[2] Xu Y., Li Z. (2020). CRISPR-Cas systems: Overview, innovations and applications in human disease research and gene therapy. Comput. Struct. Biotechnol. J..

[3] Ledford H., Callaway E. (2020). Pioneers of revolutionary CRISPR gene editing win chemistry Nobel. Nature.

[4] Vicente M.M., Chaves-Ferreira M., Jorge JM P., Proenca J.T., Barreto V.M. (2021). The Off-Targets of Clustered Regularly Interspaced Short Palindromic Repeats Gene Editing. Front. Cell Dev. Biol..

[5] Pourcel C., Touchon M., Villeriot N., Vernadet J.P., Couvin D., Toffano-Nioche C., Vergnaud G. (2020). CRISPRCasdb a successor of CRISPRdb containing CRISPR arrays and cas genes from complete genome sequences, and tools to download and query lists of repeats and spacers. Nucleic Acids Res..

[6] Parks D.H., Chuvochina M., Rinke C., Mussig A.J., Chaumeil P.A., Hugenholtz P. (2022). GTDB: An ongoing census of bacterial and archaeal diversity through a phylogenetically consistent, rank normalized and complete genome-based taxonomy. Nucleic Acids Res..

[7] Huang H., Lv W., Li J., Huang G., Tan Z., Hu Y., Ma S., Zhang X., Huang L., Lin Y. (2023). Comparison of DNA targeting CRISPR editors in human cells. Cell Biosci..

[8] Li F., Wing K., Wang J.H., Luu C.D., Bender J.A., Chen J., Wang Q., Lu Q., Nguyen Tran M.T., Young K.M. (2020). Comparison of CRISPR/Cas Endonucleases for in vivo Retinal Gene Editing. Front. Cell Neurosci..

[9] Zhang F. (2019). Development of CRISPR-Cas systems for genome editing and beyond. Q. Rev. Biophys..

[10] Mahas A., Marsic T., Lopez-Portillo Masson M., Wang Q., Aman R., Zheng C., Ali Z., Alsanea M., Al-Qahtani A., Ghanem B. (2022). Characterization of a thermostable Cas13 enzyme for one-pot detection of SARS-CoV-2. Proc. Natl. Acad. Sci. USA.

[11] Shao Z., Zhao H., Zhao H. (2009). DNA assembler, an in vivo genetic method for rapid construction of biochemical pathways. Nucleic Acids Res..

[12] Lobs A.K., Schwartz C., Wheeldon I. (2017). Genome and metabolic engineering in non-conventional yeasts: Current advances and applications. Synth. Syst. Biotechnol..

[13] Xia Y., Li Y., Shen W., Yang H., Chen X. (2023). CRISPR-Cas Technology for Bioengineering Conventional and Non-Conventional Yeasts: Progress and New Challenges. Int. J. Mol. Sci..

[14] Cardi T., Murovec J., Bakhsh A., Boniecka J., Bruegmann T., Bull S.E., Eeckhaut T., Fladung M., Galovic V., Linkiewicz A. (2023). CRISPR/Cas-mediated plant genome editing: Outstanding challenges a decade after implementation. Trends Plant Sci..

[15] Reza Khorramizadeh M., Saadat F., Verma A.S., Singh A. (2020). Chapter 8—Animal Models for Human Disease. Animal Biotechnology.

[16] Groza T., Gomez F.L., Mashhadi H.H., Munoz-Fuentes V., Gunes O., Wilson R., Cacheiro P., Frost A., Keskivali-Bond P., Vardal B. (2023). The International Mouse Phenotyping Consortium: Comprehensive knockout phenotyping underpinning the study of human disease. Nucleic Acids Res..

[17] Matveeva A., Vinogradov D., Zhuravlev E., Semenov D., Vlassov V., Stepanov G. (2023). Intron Editing Reveals SNORD-Dependent Maturation of the Small Nucleolar RNA Host Gene GAS5 in Human Cells. Int. J. Mol. Sci..

[18] Abashkin D.A., Karpov D.S., Kurishev A.O., Marilovtseva E.V., Golimbet V.E. (2023). ASCL1 Is Involved in the Pathogenesis of Schizophrenia by Regulation of Genes Related to Cell Proliferation, Neuronal Signature Formation, and Neuroplasticity. Int. J. Mol. Sci..

[19] Jahangir M., Zhou J.S., Lang B., Wang X.P. (2021). GABAergic System Dysfunction and Challenges in Schizophrenia Research. Front. Cell Dev. Biol..

[20] Bhandari A., Voineskos D., Daskalakis Z.J., Rajji T.K., Blumberger D.M. (2016). A Review of Impaired Neuroplasticity in Schizophrenia Investigated with Non-invasive Brain Stimulation. Front. Psychiatry.

[21] Liu M., Rehman S., Tang X., Gu K., Fan Q., Chen D., Ma W. (2018). Methodologies for Improving HDR Efficiency. Front. Genet..

[22] Leal A.F., Herreno-Pachon A.M., Benincore-Florez E., Karunathilaka A., Tomatsu S. (2024). Current Strategies for Increasing Knock-In Efficiency in CRISPR/Cas9-Based Approaches. Int. J. Mol. Sci..

[23] Zhang S., Shen J., Li D., Cheng Y. (2021). Strategies in the delivery of Cas9 ribonucleoprotein for CRISPR/Cas9 genome editing. Theranostics.

[24] Bloomer H., Khirallah J., Li Y., Xu Q. (2022). CRISPR/Cas9 ribonucleoprotein-mediated genome and epigenome editing in mammalian cells. Adv. Drug Deliv. Rev..

[25] Arroyo-Olarte R.D., Bravo Rodriguez R., Morales-Rios E. (2021). Genome Editing in Bacteria: CRISPR-Cas and Beyond. Microorganisms.

[26] Riesenberg S., Helmbrecht N., Kanis P., Maricic T., Paabo S. (2022). Improved gRNA secondary structures allow editing of target sites resistant to CRISPR-Cas9 cleavage. Nat. Commun..

[27] Takeuchi S., Yamamoto M., Matsumoto S., Kenjo E., Karashima M., Ikeda Y. (2022). Pinpoint modification strategy for stabilization of single guide RNA. J. Chromatogr. B Analyt Technol. Biomed. Life Sci..

[28] Rozners E. (2022). Chemical Modifications of CRISPR RNAs to Improve Gene-Editing Activity and Specificity. J. Am. Chem. Soc..

[29] Sun B., Chen H., Gao X. (2021). Versatile modification of the CRISPR/Cas9 ribonucleoprotein system to facilitate in vivo application. J. Control. Release.

[30] Westra E.R., Dowling A.J., Broniewski J.M., van Houte S. (2016). Evolution and Ecology of CRISPR. Annu. Rev. Ecol. Evol. Syst..

[31] Moasser E., Moasser A., Zaraket H. (2019). Incidence of antiviral drug resistance markers among human influenza A viruses in the Eastern Mediterranean Region, 2005-2016. Infect. Genet. Evol..

[32] Lampejo T. (2020). Influenza and antiviral resistance: An overview. Eur. J. Clin. Microbiol. Infect. Dis..

[33] Razonable R.R. (2018). Drug-resistant cytomegalovirus: Clinical implications of specific mutations. Curr. Opin. Organ. Transplant..

[34] Lan K., Luo M.H. (2017). Herpesviruses: Epidemiology, pathogenesis, and interventions. Virol. Sin..

[35] Odenwald M.A., Paul S. (2022). Viral hepatitis: Past, present, and future. World J. Gastroenterol..

[36] Cohen J.I. (2020). Herpesvirus latency. J. Clin. Investig..

[37] Niizuma K., Ogawa Y., Kogure T., Tominaga T. (2020). Case reports of latent HBV hepatitis in patients after neurosurgical treatment for hypothalamic and pituitary tumors. BMC Infect. Dis..

[38] Malahe S.R.K., van Kampen J.J.A., Manintveld O.C., Hoek R.A.S., den Hoed C.M., Baan C.C., Kho M.M.L., Verjans G. (2023). Current Perspectives on the Management of Herpesvirus Infections in Solid Organ Transplant Recipients. Viruses.

[39] Griffith B.P., Goerlich C.E., Singh A.K., Rothblatt M., Lau C.L., Shah A., Lorber M., Grazioli A., Saharia K.K., Hong S.N. (2022). Genetically Modified Porcine-to-Human Cardiac Xenotransplantation. N. Engl. J. Med..

[40] Wolff M.W., Reichl U. (2008). Downstream Processing: From Egg to Cell Culture-Derived Influenza Virus Particles. Chem. Eng. Technol..

[41] Hryhorowicz M., Lipinski D., Zeyland J. (2023). Evolution of CRISPR/Cas Systems for Precise Genome Editing. Int. J. Mol. Sci..

[42] Karpov D.S., Sosnovtseva A.O., Pylina S.V., Bastrich A.N., Petrova D.A., Kovalev M.A., Shuvalova A.I., Eremkina A.K., Mokrysheva N.G. (2023). Challenges of CRISPR/Cas-Based Cell Therapy for Type 1 Diabetes: How Not to Engineer a “Trojan Horse”. Int. J. Mol. Sci..

[43] Paschoudi K., Yannaki E., Psatha N. (2023). Precision Editing as a Therapeutic Approach for beta-Hemoglobinopathies. Int. J. Mol. Sci..

[44] Adashi E.Y., Gruppuso P.A., Cohen I.G. (2024). CRISPR Therapy of Sickle Cell Disease: The Dawning of the Gene Editing Era. Am. J. Med..

[45] Cong L., Ran F.A., Cox D., Lin S., Barretto R., Habib N., Hsu P.D., Wu X., Jiang W., Marraffini L.A. (2013). Multiplex genome engineering using CRISPR/Cas systems. Science.

[46] Mali P., Yang L., Esvelt K.M., Aach J., Guell M., DiCarlo J.E., Norville J.E., Church G.M. (2013). RNA-guided human genome engineering via Cas9. Science.

[47] Jinek M., East A., Cheng A., Lin S., Ma E., Doudna J. (2013). RNA-programmed genome editing in human cells. Elife.

